# Supplementary material for: Single-Cell Multiomics Reveals Clonal T-Cell Expansions and Exhaustion in Blastic Plasmacytoid Dendritic Cell Neoplasm
Source: Front Immunol. 2022 Mar 10;13:809414. doi: 10.3389/fimmu.2022.809414 (PMC8960171; doi:10.3389/fimmu.2022.809414)
Supplement: Supplementary file 1 [file DataSheet_1.docx]

Supplementary Material

# Supplementary Figures

##
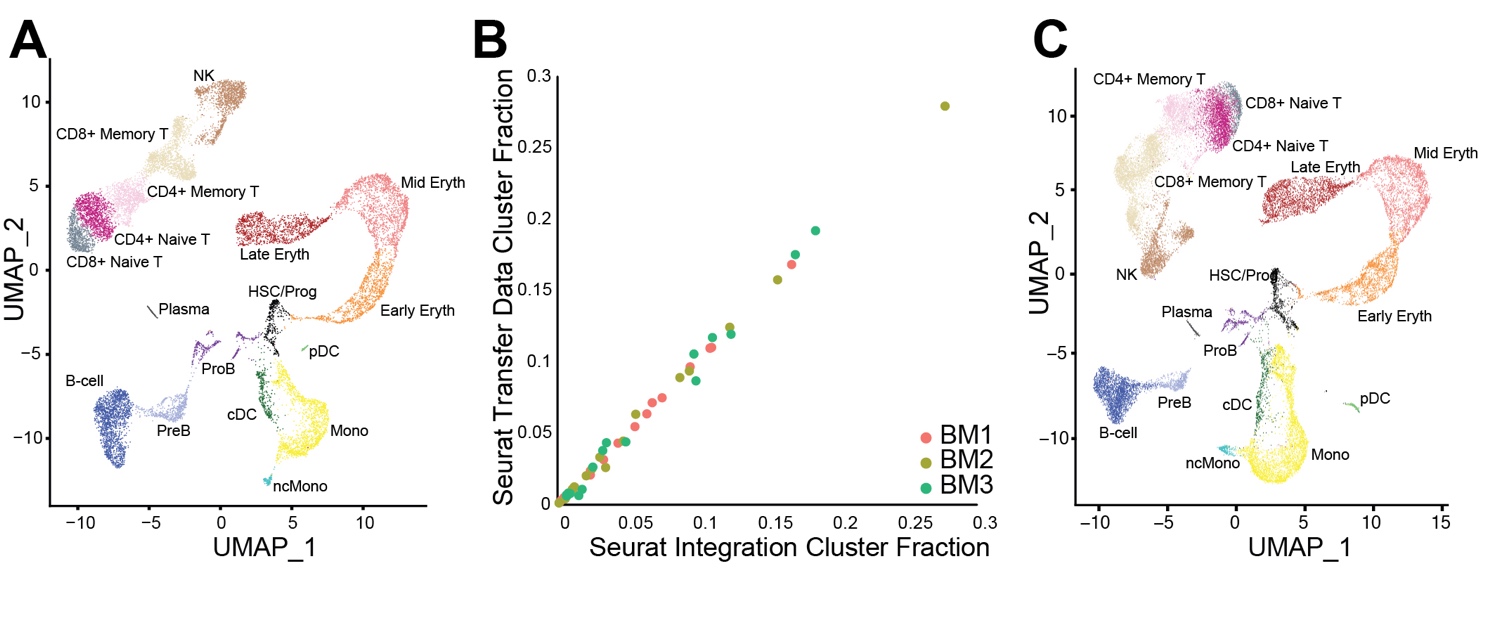


## Supplemental Figure 1: Integration and merging of healthy control samples.

(A) UMAP visualization of Seurat integrated scRNA-seq data for hematopoietic cells from BM 1-3.

(B) Plot of the fraction of each cell type from each sample as classified by Seurat Integration (x-axis) and Seurat TransferData (y-axis). Colors represent samples, where each sample has up to 17 cell types present as dots on the graph.

(C) UMAP of Seurat clustering and sub-clustering of all BM samples (BM 1-5) identified 17 clusters of cells with similar transcriptional states following merging of BM 4-5 with the BM 1-3 reference from (A). Colors for each cell type in the UMAP plots represent cell types identified in the figure legend for **Figure 1**.


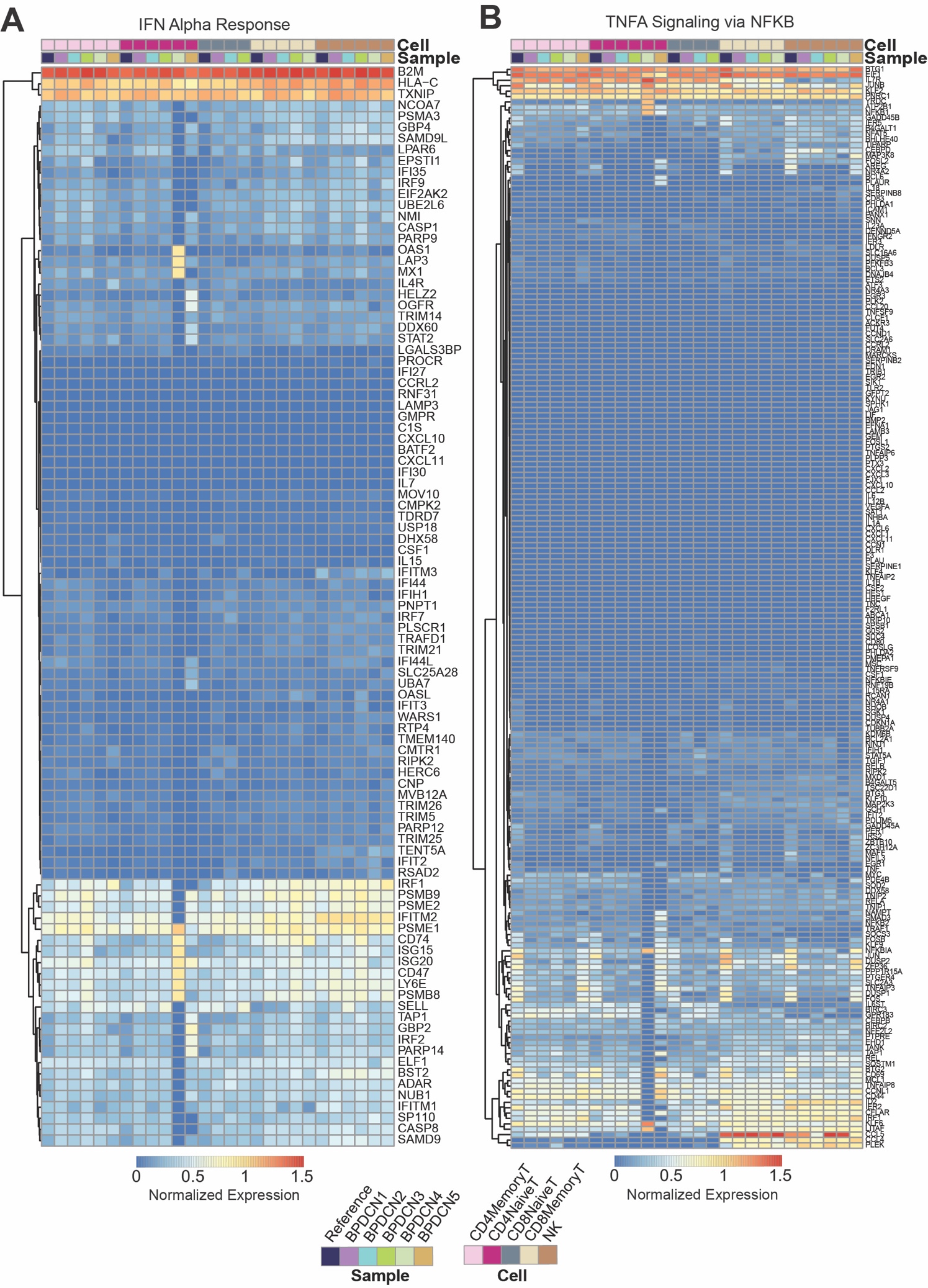


**Supplemental Figure 2: Expression of full IFNA Response and TNFA Signaling via NFKB gene sets in T and NK cells.**

(A) Heatmap shows log expression values for genes in the IFN Alpha signaling gene set (rows) for each sample and cell type (columns), clustered by row. Red indicates higher expression and blue indicates lower expression.

(B) Heatmap shows log expression values for genes in the TNFA Signaling via NFKB gene set.

**
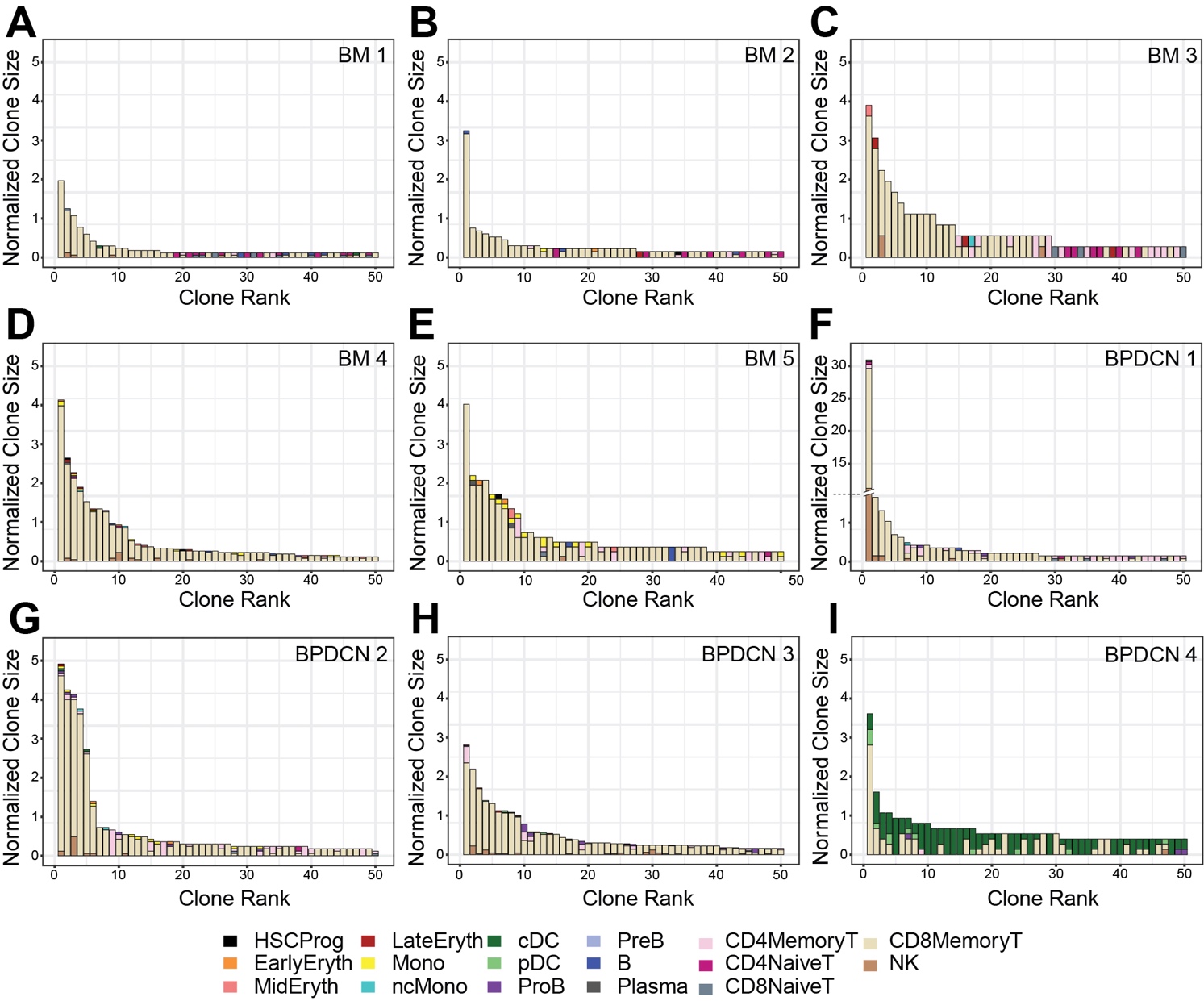
**

**Supplemental Figure 3: T-cell clonotype sizes in healthy controls and BPDCN patients.**

(A-E) Bar plots of the top 50 ranked normalized clone sizes for BM 1-5 healthy controls. The normalized clone size is the percentage of cells expressing the same TCR of all cells in which a TCR was detected. For each clone (stacked bar), the colors indicate the proportion comprised of that cell type.

(F-I) Bar plots of ranked clones for BPDCN 1-4 patient samples. Axis break in (F) is indicated by dashed line and broken bar.


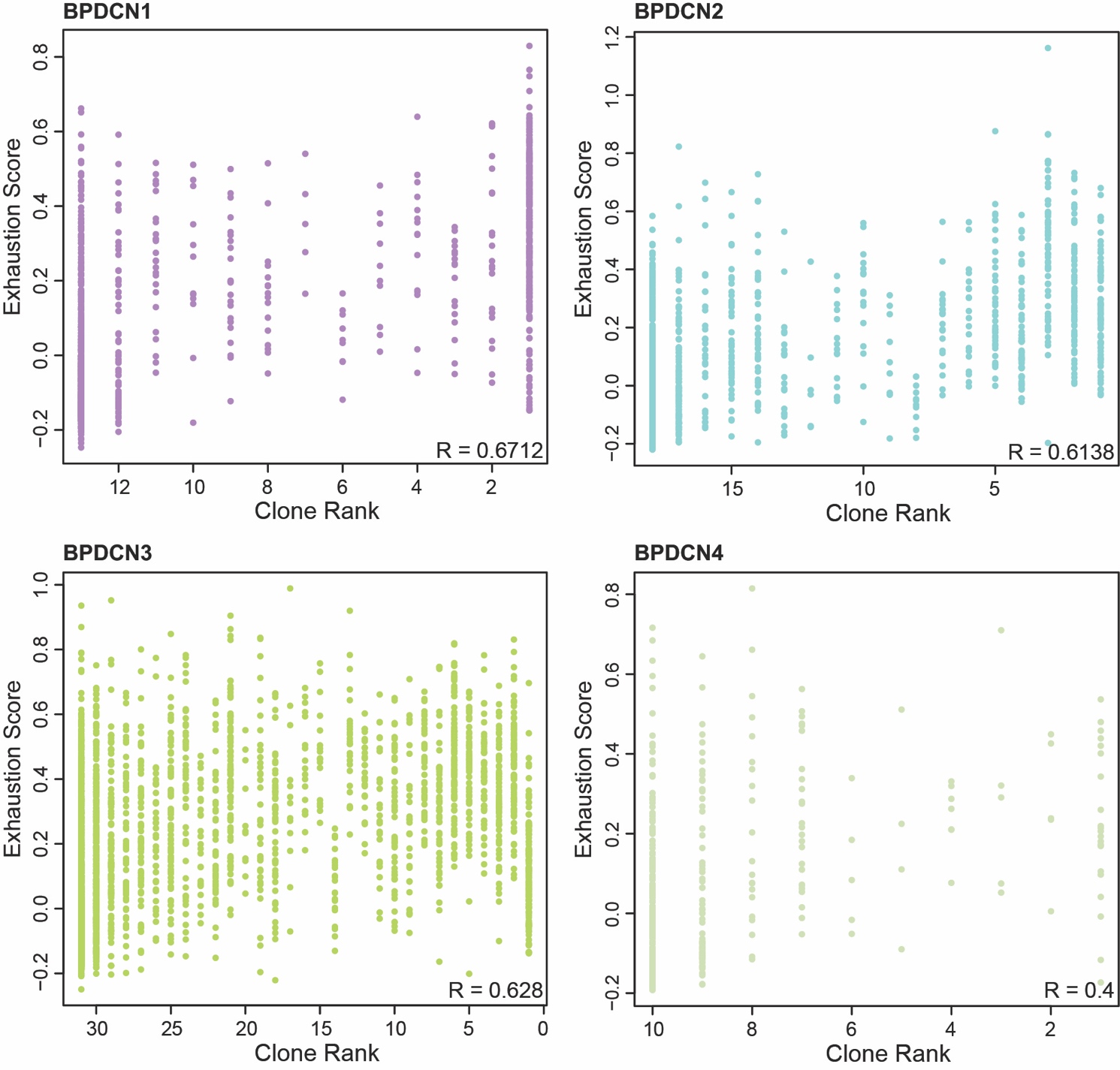


**Supplemental Figure 4: Clone size positively correlates with exhaustion score at the cell level in BPDCN T-cells.**

Correlation plots of T-cells for each of the BPDCN 1-4 samples, with each dot representing a cell. Clone rank (x-axis) of 1 means the most expanded clone of the dataset, with increasing rank following decreasing size. Exhaustion scores per cell are indicated by the y-axis. Spearman correlation values (R) for the correlation are displayed in the bottom right corner of each plot.

# Supplementary Tables

**Supplemental Table 1: Donor and patient demographics.** This table is also available as a Supplemental Excel file.

**Supplemental Table 2: Primer sequences.** This table is also available as a Supplemental Excel file.

**Supplemental Table 3: cellHarmony cell classifications.** This table is also available as a Supplemental Excel file.
